# A Descriptive Analysis of Cancer Screening Health Literacy Among Black Women Living with HIV in Baltimore, Maryland

**DOI:** 10.3928/24748307-20220616-01

**Published:** 2022-07

**Authors:** Chun-An Sun, Joyline Chepkorir, Kyra Jennifer Waligora Mendez, Joycelyn Cudjoe, Hae-Ra Han

## Abstract

**Background:**

Black women living with HIV (WLH) have the highest HIV infection rate, cervical cancer mortality, and the lowest cancer screening use compared to other groups. However, there is a gap in knowledge about cancer screening health literacy in the Black WLH population.

**Objective:**

The purpose of this study was to assess the level of cancer screening health literacy, and to identify factors associated with health literacy among Black WLH.

**Methods:**

This study used baseline data from a community-based randomized controlled trial for a health literacy intervention called CHECC-uP (community-based health literacy intervention for cancer control). We recruited a convenience sample of Black WLH (*N* = 123) who understand English and had no Pap testing in the prior 12 months. The outcome was cancer screening health literacy measured with a validated health literacy tool—Assessment of Health Literacy in Cancer Screening. Predictors included age, marital status, education, income, and insurance type. The association between cancer screening health literacy and predictors was assessed with multivariate logistic regression.

**Key Results:**

Almost one-half (49.6%) of study participants had a reading level at or below sixth grade. Older age (adjusted odds ratio [aOR] 1.05) and higher educational levels (aOR 5.13) were significantly associated with higher cancer screening health literacy among our sample of Black WLH in bivariate and multivariate analyses.

**Conclusions:**

Educational materials and other approaches to empower patients should be tested with patients who have low health literacy to ensure efficacy. [***HLRP: Health Literacy Research and Practice*. 2022;6(3):e175–e181.**]

**Plain Language Summary:**

Using a cancer screening health literacy tool, we found that about one-half of the Black WLH in the study had a reading level at or below sixth grade. Age and education level were related to their reading levels among the women. Researcher and clinicians need to test educational materials and other approaches with patients who have low health literacy to make sure they work.

According to the Centers for Disease Control and Prevention (CDC, 2020), 1.1 million people in the United States are living with HIV, and 23.5% of these are women. Women living with HIV (WLH) in the U.S. experience health disparities. For example, WLH have increased risk of cervical cancer than women in the general population due to immunosuppression and higher incidence of co-infection with the sexually transmitted virus, human papillomavirus (Shiels & Engels, 2017). Of all racial groups, Black women have the highest incidence and prevalence of HIV infection (CDC, 2019) and the highest age-adjusted mortality from cervical cancer (Howlader et al., 2020). A recently published review found that lower health literacy was associated with lower uptake of cervical cancer screening (i.e., Pap testing) (Fuzzell et al., 2021).

Health literacy is defined as the degree to which individuals have the capacity to obtain, process, and understand basic health information and services needed to make appropriate health decisions (U.S. Department of Health and Human Services, 2019). An estimated 80 million adults in the U.S. have limited health literacy levels (Hickey et al., 2018). The National Center for Education Statistics (n.d.), through the 2017 Program for the International Assessment of Adult Competencies, found that White individuals had higher numeracy, literacy, and digital problem-solving scores than Black individuals. Additionally, people with low education, income, and English proficiency, and the elderly are disproportionately affected by low health literacy (Institute of Medicine, 2004). A recent systematic review summarizing studies testing a theory-based health literacy framework also found that marital status, health insurance status, and internet use were antecedents to health literacy (Cudjoe et al., 2020).

Health literacy has been significantly associated with better Pap testing across racially diverse women who are not infected with HIV, even after controlling for individual and contextual factors (Kim & Han, 2016; Sentell et al., 2015). Within the context of HIV and Pap testing, there is limited literature addressing health literacy. Bynum et al. (2013) in a qualitative study of 145 WLH (90% Black) examined the influence of health literacy on cervical cancer knowledge and found that fewer women with low health literacy had Pap testing in the last year compared to women with high health literacy (75% versus 86%). Disparities in Pap testing and health literacy among Black WLH have been linked to structural and institutional racism, which limit educational opportunities and access to culturally appropriate health services and information, as well as contribute to health system mistrust (Davis et al., 2020; Fuzzell et al., 2021; Muvuka et al., 2020).

Previous studies examining the relationship between Pap testing and health literacy have measured health literacy using noncontext-specific instruments (Oldach & Katz, 2014). For example, commonly used instruments include the Single-Item Literacy Screener, which measures how often someone needs help reading health information (Morris et al., 2006); and the Test of Functional Health Literacy in Adults, which assesses patient's reading and comprehension of common healthcare terms (Parker et al., 1995). Failing to address health literacy in the context of cervical cancer screening may cause inaccurate understanding of health literacy needs among vulnerable populations, including Black WLH. The purpose of this study was to assess the level of cancer screening health literacy and to identify associated factors among WLH, using context-specific and non-context specific tools. Findings from this study can help identify health literacy needs of WLH in the context of cervical cancer screening.

## Methods

### Study Design and Sample

We used baseline data from a randomized controlled trial called CHECC-uP (community-based health literacy intervention for Cancer Control). Details about this intervention can be found on ClinicalTrials.gov (NCT03033888). Briefly, the CHECC-uP trial was designed to test the effects of a health literacy-focused cervical cancer prevention intervention among WLH. Inclusion criteria were (1) WLH age ≥18 years, (2) no Pap testing in the prior year at the time of enrollment (Peprah et al., 2018) as suggested by the Infectious Disease Society of America that WLH age 30 years or greater should receive annual Pap testing, then every 3 years if the results of three consecutive Pap tests are normal (Thompson et al., 2021), (3) able to understand English, and (4) own a phone. WLH with a history of hysterectomy were excluded. Eligibility criteria were assessed with self-report questions.

### Procedures

The Johns Hopkins Medicine institutional Review Board approved all study procedures. Study participants were recruited using community-based methods, which are published elsewhere (Mendez et al., 2021). In short, women were referred by community organizations, local HIV health clinics, and an HIV/AIDS research center affiliated with a university (Mendez et al., 2021). Trained research staff screened women over the phone for eligibility. Women who met eligibility criteria were scheduled for baseline visits at community locations. Every woman provided written informed consent to participate. Trained research staff collected data using an online Qualtrics survey, which was administered on an encrypted and password-protected desktop or laptop computers (except for two women who preferred a paper-pencil survey). Each participant received a $20 gift card.

### Measurement

Individual characteristics, including age, education, health insurance, marital status, and subjective income comfort level, were collected using a questionnaire created for study purposes. Of note, a direct question about participant's income was purposely avoided following a suggestion from the community advisory board as a method to minimize missing responses. Health literacy was measured using two validated health literacy assessment tools: Rapid Estimate of Adult Literacy in Medicine-Revised (REALM-R) and the Assessment of Health Literacy in Cancer Screening (AHL-C).

REALM-R is a shortened version of the REALM, one of the most widely used noncontext-specific health literacy assessment tools that measures an individual's ability to pronounce 66 common medical words. REALM-R includes 11 items, three calibration nonscored items and eight scored items. Each correctly pronounced item is scored as 1, and total scores range from 0 to 8. REALM-R was validated with REALM (Pearson's correlation coefficient = 0.72), where a total raw score of six corresponds to a sixth grade reading level (Bass III et al., 2003). We chose REALM-R to minimize response burden.

AHL-C is a 52-item validated instrument assessing cancer screening health literacy. AHL-C consists of five sub-scales: reading test, numeracy, familiarity, comprehension, and health navigational literacy. AHL-C has been validated in Korean American immigrant women (Han et al., 2014). For this study, we used the reading test on AHL-C, which includes 12 commonly used medical words in the context of women's cancer screening **(Table A)**. As is the case with REALM-R, each correctly pronounced word on the AHL-C reading test is scored as 1, with total scores ranging from 0 to 12. To properly estimate the grade range, we used a receiver operating characteristic (ROC) analysis between REALM-R and AHL-C. We determined that a person had a ≤6th-grade reading level if the AHL-C reading test scores ranged from 0 to 8 (sensitivity: 85.5%, specificity: 85.3%).

**Table A:**
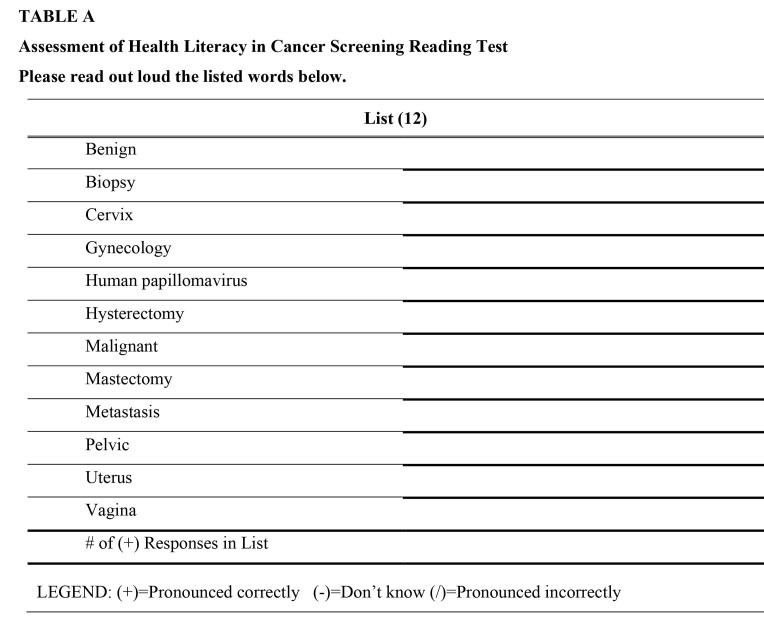
Assessment of Health Literacy in Cancer Screening Reading Test Please read out loud the listed words below.

**List (12)**	
Benign	_______________________________
Biopsy	_______________________________
Cervix	_______________________________
Gynecology	_______________________________
Human papillomavirus	_______________________________
Hysterectomy	_______________________________
Malignant	_______________________________
Mastectomy	_______________________________
Metastasis	_______________________________
Pelvic	_______________________________
Uterus	_______________________________
Vagina	_______________________________
# of (+) Responses in List	_______________________________

LEGEND: (+)=Pronounced correctly (−)=Don't know (/)=Pronounced incorrectly

### Analysis

We used descriptive statistics to summarize sample characteristics. For health literacy measurements, we assumed a missing item was pronounced incorrectly, and thus, assigned a 0 for that item. First, we used simple logistic regression to examine bivariate associations between each predictor and the outcome, cancer screening health literacy. Then, we used a multiple logistic regression model including all predictor variables. Our selection of predictor variables was guided by a recent systematic review in which age, marital status, education, income, health insurance status, race and ethnicity, general literacy and English proficiency, and internet use were antecedents to health literacy (Cudjoe et al., 2021). The parent study included the following variables: age, marital status, education, income, type of insurance, and race and ethnicity. We did not include race and ethnicity and insurance type in regression models because our sample was 100% Black and most (95.9 %) participants had Medicare, Medicaid, or both. We dichotomized education at its median, and other variables were operationalized based on previous health literacy literature (Han et al., 2011; Han et al., 2014). We grouped Medicaid and Medicare together, as all enrolled participants were younger than age 65 years and most Medicare beneficiaries with HIV are dual eligible for Medicare and Medicaid (Kaiser Family Foundation, 2016). All statistical analyses were conducted with Stata 16.0. Statistical significance was determined at *p* < .05.

## Results

### Demographics of Study Participants

A total of 123 WLH completed the baseline survey and were included in this study (see **Table 1** for sample characteristics). The mean age was 51.9 years (range: 22-77, standard deviation [*SD*] 9) and all enrolled women were Black. Most participants were not married/partnered (77.1%) and had an education level ≥ high-school graduate (58.5%). More than one-half of participants self-rated their income levels as more than okay (64.7%) and did not work (e.g., were unemployed, retired, or disabled) at the time of data collection (90.2%). Nearly all participants (95.9%) had Medicare, Medicaid, or both.

**Table 1:**
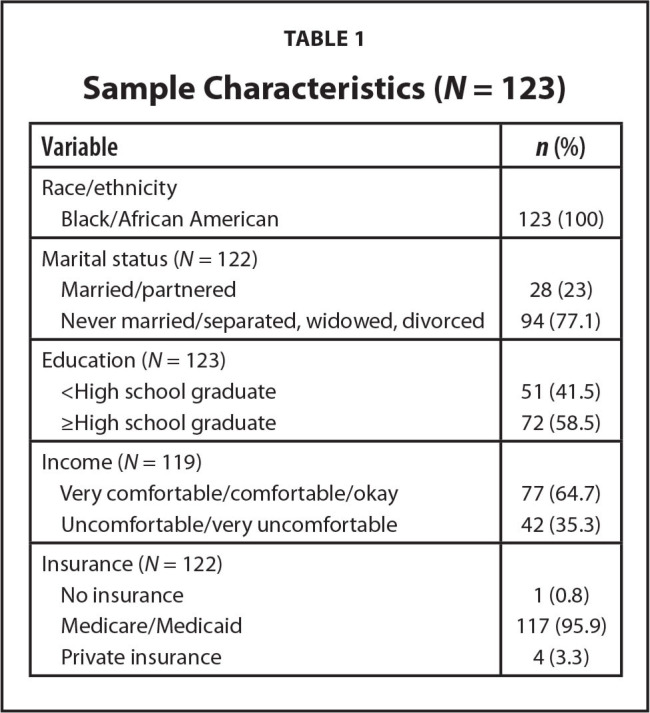
Sample Characteristics (*N* = 123)

**Variable**	***n* (%)**

Race/ethnicity	
Black/African American	123 (100)

Marital status (*N* = 122)	
Married/partnered	28 (23)
Never married/separated, widowed, divorced	94 (77.1)

Education (*N* = 123)	
<High school graduate	51 (41.5)
≥High school graduate	72 (58.5)

Income (*N* = 119)	
Very comfortable/comfortable/okay	77 (64.7)
Uncomfortable/very uncomfortable	42 (35.3)

Insurance (*N* = 122)	
No insurance	1 (0.8)
Medicare/Medicaid	117 (95.9)
Private insurance	4 (3.3)

### General Health Literacy Versus Cancer Screening Health Literacy

Nine participants had one-item missing on REALM-R. The average score of REALM-R was 5.7 (*SD* 2.4), with 61 WLH (49.4%) categorized as having a reading level of sixth grade or lower. For the AHL-C reading test, the average score was 8.1 (*SD* 3.2) and four participants missed one item. Using the cut-off point set by the ROC analysis, 61 WLH (49.6%) were determined to have a cancer screening-specific reading level of sixth grade or lower.

### Results of Logistic Regression Models

The coefficients of simple and multiple logistic regression models are presented in **Table 2**. Higher education attainment had the strongest association with cancer screening health literacy in both bivariate (odds ratio [OR]: 5.29, 95% confidence interval [CI] 2.41, 11.60, *p* < .001) and multivariate logistic regression models. Specifically, the odds of having a reading level above sixth grade among women with high education levels (high school or greater) was 5.13 times the odds (95% CI 1.27, 20.66, *p* < .05) of having a reading level above sixth grade among women with low education levels (<high school), after controlling for all covariates. Older age was also statistically significant in the bivariate (OR: 1.05, 95% CI 1.00, 1.09, *p* < .05) and multivariate logistic regression models (aOR 1.05, 95% CI 1.00, 1.09, *p* < .05). Marital status and income were not significantly associated with cancer screening health literacy.

**Table 2:**
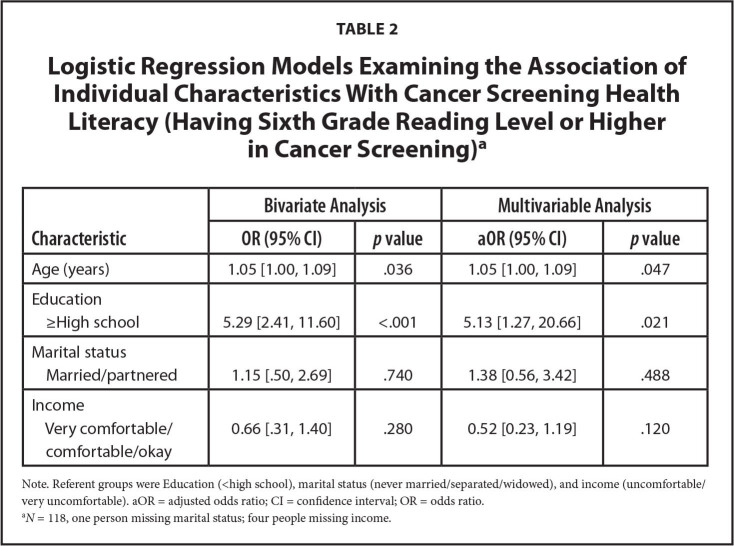
Logistic Regression Models Examining the Association of Individual Characteristics With Cancer Screening Health Literacy (Having Sixth Grade Reading Level or Higher in Cancer Screening)^a^

**Characteristic**	**Bivariate Analysis**		**Multivariable Analysis**	

	**OR (95% CI)**	***p* value**	**aOR (95% CI)**	***p *value**

Age (years)	1.05 [1.00, 1.09]	.036	1.05 [1.00, 1.09]	.047

Education				
≥High school	5.29 [2.41, 11.60]	<.001	5.13 [1.27, 20.66]	.021

Marital status				
Married/partnered	1.15 [.50, 2.69]	.740	1.38 [0.56, 3.42]	.488

Income				
Very comfortable/comfortable/okay	0.66 [.31, 1.40]	.280	0.52 [0.23, 1.19]	.120

Note. Referent groups were Education (<high school), marital status (never married/separated/widowed), and income (uncomfortable/very uncomfortable). aOR = adjusted odds ratio; CI = confidence interval; OR = odds ratio.

a *N* = 118, one person missing marital status; four people missing income.

## Discussion

This study examined cancer screening health literacy and its association with key demographic characteristics among WLH who did not have Pap testing in the prior 12 months. We found that one-half of participants had a sixth grade reading level or lower, and that higher education levels and older age were associated with higher cancer screening health literacy. Whereas previous research involving people living with HIV used noncontext-specific single-item literacy screening (Bynum et al., 2013), our study adds to the current literature by providing information about cancer screening-specific health literacy among WLH who are disproportionately affected by cervical cancer.

One-half of the WLH in our sample had a cancer screening reading level at or below sixth grade, indicating that WLH might have decreased capacity to take responsibility for their health (Sørensen et al., 2012) and that WLH may struggle with most written health information (U.S. Department of Health and Human Services, 2008). Using the same assessment tool (i.e., AHL-C), Cudjoe et al. (2021) found a similarly high rate of low health literacy (47%) among African immigrant women. According to the National Assessment of Adult Literacy, a nationally representative assessment of English literacy among more than 19,000 Americans age 16 years and older, more than one-third of the U.S. population had basic or below basic health literacy skills, requiring low literacy education materials composed mainly of audio or video materials (Kutner et al., 2006). This has important implications for health care providers, who should ensure their health communication and written educational materials are simplified to a reading level at or below sixth grade to improve patient understanding and reduce health disparities (Brega et al., 2015).

Our study indicates that using AHL-C among WLH can appropriately identify individuals with low health literacy levels. Two studies among people living with HIV found only 12% to 38% who had a lower health literacy using non-cancer screening measurements (Bynum et al., 2013; Waite et al., 2008), which may significantly underestimate the true population proportion of WLH with low health literacy. This is important because women with lower health literacy levels receive less Pap testing (Bynum et al., 2013) and are less likely to follow up for abnormal Pap smears (Lindau et al., 2006). Given the cervical cancer disparities among WLH, their increased risk for cervical cancer (Shiels & Engels, 2017), and the multi-dimensional aspects of health literacy (Baker, 2006), future research should include WLH from diverse racial/ethnic backgrounds with larger sample sizes to adequately assess the level of cancer screening health literacy with context-specific tools.

Regarding the antecedents of health literacy as identified in a systematic review (Cudjoe et al., 2021), we found that older age and higher educational level were significantly associated with cancer screening health literacy in our study sample. One-year increase in age was associated with a 5% increase in the odds of having cancer screening health literacy. This finding contrasts with that in the general population, for whom older age has been associated with lower health literacy (Cudjoe et al., 2021; Kutner et al., 2006). It is unclear why there was a reverse correlation between age and cancer screening health literacy among WLH in our sample. A possible explanation is that older women in our sample may have had more encounters with the health care system for Pap testing than younger women resulting in higher cancer screening health literacy. A similar trend has been observed in persons with chronic illnesses, such as type 2 diabetes (Chin et al., 2021) and hypertension (Duwe et al., 2018). These studies report that years of illness among people with chronic illnesses have enhanced their health knowledge and may facilitate their comprehension of medical terms (i.e., health literacy) (Chin et al., 2021; Duwe et al., 2018). Future research is warranted to systematically investigate how age influences cancer screening health literacy among WLH.

Education has been a strong predictor of health literacy in both general populations (Cudjoe et al., 2021; Kutner et al., 2006) and people living with HIV (Waite et al., 2008). We also found that WLH with a higher level of education (≥high school) had more than 5 times the odds of having high cancer screening reading level, controlling for other demographic characteristics. The participants in this study had a higher percentage of women with lower than a high school education (41.5%) compared to the Women's Interagency HIV Study (the oldest ongoing cohort study of WLH), in which 24% to 29% had attained less than high school education across five waves (Adimora et al., 2018). This might reflect the educational disparities in Baltimore, Maryland (Holleman, 2019). Also, higher HIV prevalence has been observed among people with lower educational levels (Denning & DiNenno, 2019), who were also less likely to complete Pap testing (Fletcher et al., 2014; Murfin et al., 2020). These results highlight the importance of bridging the educational attainment gap by providing education materials with appropriate reading levels for WLH, especially those belonging to racial minority groups.

This study addresses one of many barriers to Pap testing, which is health literacy. Besides low health literacy, transportation and logistical barriers have also contributed to lower uptake in Pap testing (Fuzzell et al., 2021). Additionally, racial and ethnic minorities (443 uninsured Black and Hispanics in Texas) were more likely to self-identify that lack of knowledge was a barrier to complete Pap testing (Akinlotan et al., 2017). To better enhance Pap testing utilization and further reduce cervical cancer disparities among minority WLH, intersectionality of those factors needs to be considered.

## Limitations

Several limitations should be acknowledged. This study only used the reading subscale of AHL-C to estimate the grade range of the reading level. As health literacy is a multidimensional concept, this article was unable to provide a comprehensive assessment of participants' health literacy. This study was cross-sectional; thus, causality cannot be assumed when addressing the relationships between demographics and cancer screening health literacy. Likewise, we used a convenience sample of 123 WLH in Baltimore, Maryland, which may limit the generalizability of study findings to inner city WLH. Nevertheless, our study offers novel insights on inner city, Black WLH, who are underresearched (Mendez et al., 2021).

Additionally, the current study was a secondary analysis, and the original sample size calculation was based on the parent study. It is possible that we did not find significant associations between health literacy and marital status, income, and insurance type due to insufficient statistical power. The parent study included only women who self-reported speaking and understanding English. This question was included to ensure women can comprehend intervention materials in English. As only one woman was excluded from the study due to this question (Spanish-speaking), we do not believe this was a study limitation that could have influenced outcomes.

## Conclusion

Advances in HIV treatment has made it a manageable, chronic disease and more people living with HIV survive to older ages (Deeks et al., 2013). Yet, WLH continue to experience higher cervical cancer incidence and mortality with less-than-optimal Pap testing uptake. Our finding that about one-half of Black WLH in the study sample had a reading level at or below sixth grade suggests that educational materials and other approaches to empower patients should be used with patients who have low literacy to ensure efficacy. It is also important to examine how age may shape cancer screening health literacy among WLH.
